# Static and Dynamic Changes in Local Brain Connectivity in Unilateral Sudden Sensorineural Hearing Loss

**DOI:** 10.3390/bioengineering12060619

**Published:** 2025-06-05

**Authors:** Junchao Zeng, Jing Li, Bo Liu, Qun Yu, Ziqiao Lei, Fan Yang, Mingyue Ding, Wenliang Fan

**Affiliations:** 1 Medical Ultrasound Laboratory, Department of Biomedical Engineering, College of Life Science and Technology, Advanced Bio-Medical Imaging Facility, Huazhong University of Science and Technology, Wuhan 430074, China; d202380995@hust.edu.cn; 2 Department of Radiology, Union Hospital, Tongji Medical College, Huazhong University of Science and Technology, Wuhan 430022, China; lijing80603@163.com (J.L.); boqun_002@163.com (Q.Y.); ziqiao_lei@hust.edu.cn (Z.L.); fyang@hust.edu.cn (F.Y.); 3 Hubei Provincial Clinical Research Center for Precision Radiology & Interventional Medicine, Wuhan 430022, China; 4 Hubei Province Key Laboratory of Molecular Imaging, Wuhan 430022, China; 5 Department of Otorhinolaryngology Head and Neck Surgery, Union Hospital, Tongji Medical College, Huazhong University of Science and Technology, Wuhan 430022, China; liuboent@hust.edu.cn

**Keywords:** unilateral sudden sensorineural hearing loss, local brain connectivity, dynamic local metric, neuroimaging biomarkers

## Abstract

Unilateral sudden sensorineural hearing loss (SSHL) presents substantial clinical challenges owing to its abrupt onset and multifactorial, poorly understood etiology. This study investigates the static and dynamic changes in local brain connectivity using regional homogeneity (ReHo) analyses in 102 SSHL patients and 73 healthy controls. A static ReHo analysis reveals pronounced disruptions in local synchronization within motor and cognitive-related brain regions in SSHL patients compared to controls. A dynamic ReHo analysis uncovers increased temporal variability, particularly in frontal regions, indicating potential adaptive neural plasticity to auditory deficits through enhanced neural plasticity. The correlation analyses further associate these neural changes with clinical parameters, highlighting the significant positive correlations between static ReHo in the left precentral gyrus and tinnitus severity (R = 0.39, *p* < 0.001), as well as the negative correlations between dynamic ReHo in the middle frontal gyrus and the duration of hearing loss (R = −0.35, *p* < 0.001). These findings underscore the complex interplay between static neural dysregulation and dynamic adaptive mechanisms in the pathophysiology of SSHL. Emphasizing dynamic metrics, our study provides a novel temporal perspective on how the brain reorganizes in response to acute sensory loss.

## 1. Introduction

Sudden sensorineural hearing loss (SSHL) represents a critical otological emergency, defined by an abrupt decrease of at least 30 dB in auditory threshold across three contiguous frequency bands, typically manifesting within a 72 h timeframe [1,2]. It is typically unilateral, with bilateral involvement documented in fewer than 5% of cases, and can significantly impact a patient’s quality of life [1,2]. The reported incidence of SSHL ranges from 5 to 27 per 100,000 individuals, with a higher incidence observed in individuals aged 50 to 60 years, and its annual incidence continues to rise [2,3,4]. Various potential causes of SSHL have been suggested, including tumors, vascular disorders, inner ear membrane rupture, viral or bacterial infections, and autoimmune diseases [5,6,7,8,9]. However, the etiopathogenesis of more than two-thirds of SSHL cases remains undefined, collectively categorized as idiopathic. Beyond impacting the inner ear’s structure and function, such as auditory nerve fibers, cochlear hair cells, and the membranous labyrinth, SSHL can also lead to central nervous system symptoms, including cognitive impairments, depressive symptomatology, and anxiety disorders. These central symptoms significantly compromise the quality of life for individuals affected by the condition [2,10]. Therefore, a comprehensive evaluation of brain functions is essential in order to elucidate the fundamental pathophysiological mechanisms of SSHL and facilitate the formulation of efficacious therapeutic interventions.

Functional magnetic resonance imaging (fMRI) has profoundly advanced the scientific community’s comprehension of the brain’s functional architecture in various neurological and otologic conditions, including SSHL [11,12]. The resting-state fMRI has been particularly useful, allowing researchers to examine intrinsic brain activity through the quantification of spontaneous low-frequency oscillations in the blood-oxygen-level-dependent (BOLD) signal. Previous studies using the resting-state fMRI in SSHL patients have identified alterations in brain regions associated with auditory processing, including but not limited to the primary auditory cortex and related networks [13,14,15,16]. The existing research has documented modifications in large-scale brain networks, including the dorsal attention network and the default mode network, in individuals with hearing loss [17]. These extensive network alterations may potentially influence local connectivity disruptions, such as those reported in the superior temporal gyrus and inferior parietal lobule [18]. This interplay highlights the necessity of examining both global and local brain dynamics in the context of SSHL. Previous investigations have predominantly centered on the static and global metrics of brain connectivity, yielding valuable insights into the overall changes in brain function associated with SSHL. However, these static and global approaches have limitations, as they only offer a snapshot of brain activity over the entire scanning period and do not capture the dynamic nature of neural activity, which can vary significantly over time [19,20]. Moreover, they often overlook local brain connectivity, which is crucial for understanding the fine-grained functional reorganization occurring in specific brain regions affected by SSHL, as previously hypothesized in models of sensory deprivation and neuroplasticity [21].

Quantifying temporal dynamics provides critical insights into the oscillatory patterns of neural activity across sequential time domains [22,23]. Contemporary investigations have increasingly postulated that dynamic regional homogeneity (ReHo) analysis presents a methodology for quantifying modifications in the temporal heterogeneity of local brain connectivity across multiple pathological conditions. Through the implementation of dynamic ReHo analysis, this study demonstrates that subjects with various neurological and psychiatric conditions demonstrate significant temporal variability in local connectivity, which static measures fail to capture [24,25,26]. In SSHL research, a dynamic temporal variability analysis offers the potential to uncover additional pathological alterations linked to SSHL by examining the time-varying aspects of brain activity [14]. This approach can provide novel insights into the adaptive processes and compensatory mechanisms in the brain following sudden auditory deprivation, such as cross-modal plasticity and sensory compensation.

While prior rs-fMRI studies have identified disruptions in the primary auditory cortex and auditory networks in SSHL, the majority focused on static functional connectivity or global network metrics, overlooking the temporal dynamics of local brain activity. For instance, a recent study on functional connectivity gradients [27] revealed prefrontal–temporal reorganization linked to cognitive symptoms, providing a global perspective. However, the local-scale temporal variability of neural activity—critical for understanding adaptive plasticity—remains uninvestigated. Dynamic ReHo, which quantifies the moment-to-moment fluctuations in regional synchronization, has shown promise in capturing state-dependent neural changes in disorders like vestibular migraine and schizophrenia [24,25]. Here, we hypothesize that SSHL patients exhibit abnormal static ReHo in the motor/cognitive regions and increased dynamic ReHo in the frontal lobes, reflecting baseline dysregulation and compensatory plasticity, respectively.

Given the limitations of previous studies that have primarily focused on the static and global measures of brain connectivity, our research hypothesizes that SSHL patients exhibit significant changes in both static and dynamic local brain connectivity. We propose that these changes are particularly evident in brain regions associated with auditory processing and cross-modal plasticity. By investigating the dynamic aspects of ReHo, we aim to capture the temporal variability and adaptability of local brain connectivity that static analyses may overlook.

The present study encompasses four distinct objectives: (1) to characterize static ReHo alterations in brain regions intricately engaged in auditory information processing among unilateral SSHL patients; (2) to investigate dynamic ReHo changes and identify brain regions exhibiting significant temporal variability; (3) to correlate ReHo findings with clinical parameters, such as the degree of hearing loss and duration of illness; and (4) to provide a comprehensive elucidation of the neural substrates underpinning SSHL. By addressing these objectives, this study aims to advance our knowledge of SSHL and potentially inform the development of targeted therapeutic strategies.

## 2. Materials and Methods

### 2.1. Subjects

A total of 191 subjects, comprising 107 participants with unilateral SSHL and 84 age- and sex-matched healthy controls (HC), were recruited from Wuhan Union Hospital. All enrolled subjects exhibited right-handed laterality. Pure-tone audiometry was conducted using seven different octave frequencies (0.125, 0.25, 0.5, 1, 2, 4, and 8 kHz) to measure the pure tone average (PTA) and ascertain the hearing level. The diagnosis of SSHL was corroborated in accordance with the diagnostic criteria stipulated by the Clinical Practice Guideline [10]. The exclusion criteria included (1) the presence of neurological disorders (such as acoustic neuroma, brain tumors, and trauma), psychiatric disorders (such as depression and insomnia), or other otolaryngological conditions (such as otitis media, Meniere’s disease, and pulsatile tinnitus); and (2) having claustrophobia or cardiac pacemakers. The duration of hearing loss was defined as the time interval (in days) between symptom onset and MRI scanning, reflecting the acute/subacute phase of SSHL. In consideration of the high prevalence of tinnitus and/or vertigo in SSHL cases, tinnitus severity was assessed via the Tinnitus Handicap Inventory (THI), a 25-item self-report questionnaire which measures the level of handicap caused by tinnitus on a scale from 0 to 100 [28].

This study received approval from the medical ethics committee of the Union Hospital of Tongji Medical College of Huazhong University of Science and Technology. All study participants were fully apprised of the research objectives and furnished written informed consent in strict adherence to Chinese legal statutes.

A total of 191 subjects were initially recruited, including 107 unilateral SSHL patients and 84 age- and sex-matched healthy controls (HCs). After excluding participants with excessive head motion or neurological/psychiatric disorders, the final sample comprised 102 SSHL patients (52 male/50 female; age = 38.89 ± 12.11 years; education = 12.67 ± 3.20 years) and 73 HCs (37 male/36 female; age = 38.01 ± 16.52 years; education = 12.74 ± 2.93 years). All participants were right-handed. Pure-tone audiometry (PTA) was measured as the average hearing threshold across seven frequencies (0.125–8 kHz) in the affected ear for SSHL patients or the better ear for HCs, following clinical guidelines. Demographic and clinical data for the subjects are presented in Table 1. No significant differences were found between the SSHL patients and HC subjects regarding gender, age, or education level. The mean duration of 9.03 days indicates that most participants were scanned within the first two weeks of symptom onset, a critical period for capturing acute neural plasticity.

### 2.2. Data Acquisition

A 3T magnetic resonance imaging (MRI) system (Siemens Trio Tim, Erlangen, Germany) equipped with a 12-channel phased-array head coil was employed to obtain anatomical and functional images. All patients underwent imaging before receiving any drug treatment. During the imaging sessions, patients were instructed to remain still with their eyes closed and to wear noise-canceling headphones to minimize noise. A foam cushion was deployed to mitigate cephalic movements and motion-related artifacts.

Anatomical neuroimages were acquired via a three-dimensional high-resolution T1-weighted magnetization-prepared rapid acquisition gradient echo (MP-RAGE) sequence, configured with the following technical parameters: repetition time (TR) = 2250 ms, echo time (TE) = 2.26 ms, inversion time (TI) = 900 ms, flip angle = 9°, voxel size = 1.0 × 1.0 × 1.0 mm^3^, field of view (FOV) = 256 mm × 256 mm, slice thickness = 1.00 mm, and 176 sagittal slices covering the entire brain. Functional images were acquired using a gradient-echo-type echo planar imaging (EPI) sequence with the following parameters: TR = 2000 ms, TE = 30 ms, flip angle = 90°, voxel size = 3.0 × 3.0 × 3.0 mm^3^, and FOV = 200 mm × 200 mm, resulting in 240 images.

Furthermore, a T2-weighted imaging sequence was implemented to evaluate the structural and functional integrity of the peripheral auditory system, configured with the subsequent technical specifications: TR = 1000 ms, TE = 132 ms, slice thickness = 0.5 mm, slice number = 64, flip angle = 120°, FOV = 200 mm × 200 mm, and averages = 2. Two board-certified radiologists conducted independent evaluations of MR images to identify pathological entities, including but not limited to otitis media, acoustic neuroma, and intracranial neoplasms. Subjects demonstrating any of these abnormalities were excluded from subsequent quantitative analyses.

### 2.3. Data Preprocessing

The Brain Imaging Data Processing and Analysis (DPABI, v7.0) tool, based on the Statistical Parametric Mapping (SPM12) toolkits, was employed for the preprocessing of the resting-state BOLD data [29]. The initial 10 volume acquisitions were excluded to mitigate T1 equilibrium effects, leaving 230 time points for analysis. Slice-timing correction was performed to account for different slice acquisition times, and realignment correction was applied to address head motion using a six-parameter rigid-body transformation. Nuisance covariates, encompassing linear trends, white matter signal, cerebrospinal fluid signal, and Friston 24 head motion parameters were statistically regressed from the functional signal. The functional images were coregistered with the corresponding structural images to align anatomical and functional data. The structural images were then segmented and normalized to the standard Montreal Neurological Institute (MNI) template using the Diffeomorphic Anatomical Registration Through Exponentiated Lie algebra (DARTEL) tool. Following normalization, linear trends were removed, and temporal bandpass filtering (0.01–0.08 Hz) was implemented to attenuate low-frequency drift and high-frequency noise. Participants with head motion exceeding 1.5 mm in displacement, 1.5° in rotation, or with mean frame-wise displacement (FD) values exceeding 0.2, as computed via the Jenkinson algorithm, were excluded from subsequent analyses.

### 2.4. Static and Dynamic ReHo Calculation

Static ReHo was computed using Kendall’s coefficient of concordance (KCC) via the DPABI software, which was employed to quantify the similarity between a voxel’s time-series data and those of its 27 adjacent voxels. Subsequent to group-level analysis, the derived ReHo data were normalized via Fisher’s r-to-z transformation to approximate a Gaussian distribution. A 6 mm full width at half-maximum (FWHM) Gaussian kernel was subsequently employed for spatial smoothing of the functional images.

**Figure 1:**
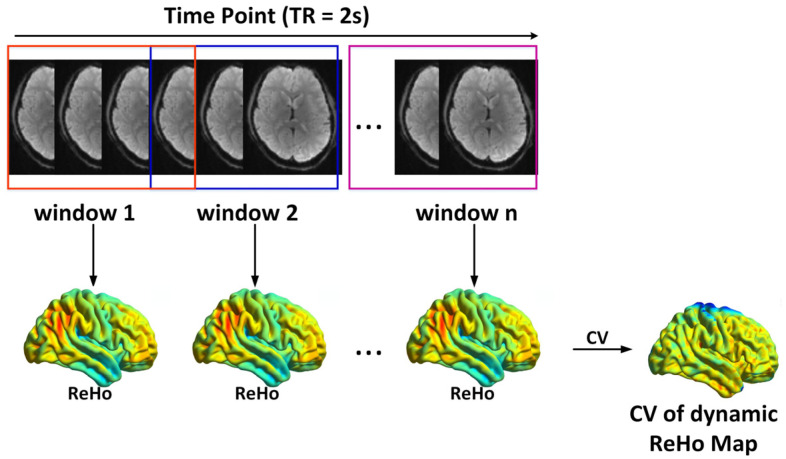
The flow chart of dynamic analysis of regional brain connectivity.

Dynamic ReHo was calculated using a sliding window approach implemented in DPABI [29], as illustrated in Figure 1. The temporal window duration represents a pivotal parameter in the computation of resting-state neurodynamics. A shorter window length can increase the risk of introducing spurious fluctuations in dynamic ReHo [22], while a longer window length may obscure the accurate depiction of temporal variability [22,30]. To avoid spurious fluctuations, the window length was set to 50 TRs (100 s), which is longer than the inverse of the minimum frequency of the time series (1/fmin) [14,22,30]. The window was shifted by 5 TRs (10 s) to capture temporal variability in ReHo, resulting in 37 windows for each participant.

The time-series data were partitioned into discrete temporal windows, upon which ReHo analyses were subsequently performed. The dynamic ReHo were represented by the coefficient of variation of the static ReHo calculated from different time windows. (CV, coefficient of variation.)

The dynamic ReHo was estimated by calculating CV of the ReHo through the *n* windows (*n* = 37) at each voxel, thereby generating subject-specific dynamic ReHo maps. Here, we defined the CV of a voxel i as follows:

(1) $$CV\left(i\right)=\frac{\sqrt{\sum_{t=1}^{n}{\left(x_{t}-x_{mean}\right)}^{2}/n}}{x_{mean}}$$

where $x_{t}$ is ReHo score of voxel i over time window *t* (*t* = 1, 2, …, *n*) and $x_{mean}$ is mean score of $x_{t}$ across n windows.

The CV maps were then z-standardized and smoothed with a 6 mm FWHM Gaussian kernel [14,31] for further statistical analysis.

### 2.5. Statistical Analysis

To characterize the spatial distribution pattern of ReHo, group means of static and dynamic ReHo were computed. A mass univariate general linear model (GLM) analysis was performed to assess group differences in static and dynamic ReHo between SSHL and HC groups, incorporating multiple regression at each voxel to control for covariates (age, sex, mean frame-wise displacement, education level, and gray matter volume). Voxel-level significance was set at *p* < 0.001, with cluster-level correction using Gaussian random field (GRF) at *p* < 0.01 implemented in DPABI, which are widely validated for such corrections. Covariates included age, sex, mean FD, education, and gray matter volume [14].

In order to elucidate the correlations between neuroimaging findings and clinical attributes of SSHL, comprehensive Pearson correlation analyses were performed between brain regions with abnormal activity and clinical characteristics of SSHL. Correlation analyses were focused on brain regions with significant group differences (preselected ROIs), reducing the need for global multiple comparison correction. Binary logistic regression was deployed to quantify the predictive probability of brain regions exhibiting altered ReHo and dynamic ReHo separately, as well as the predictive probability of combined brain regions with altered static ReHo and dynamic ReHo for the diagnosis of SSHL. Receiver operating characteristic (ROC) curves were constructed by leveraging the predictive probability as a covariate. Areas under the curve (AUCs) were used to evaluate the diagnostic value of the two metrics individually and in combination [32].

## 3. Results

### 3.1. Spatial Distribution and Group Differences of Static ReHo

**Figure 2:**
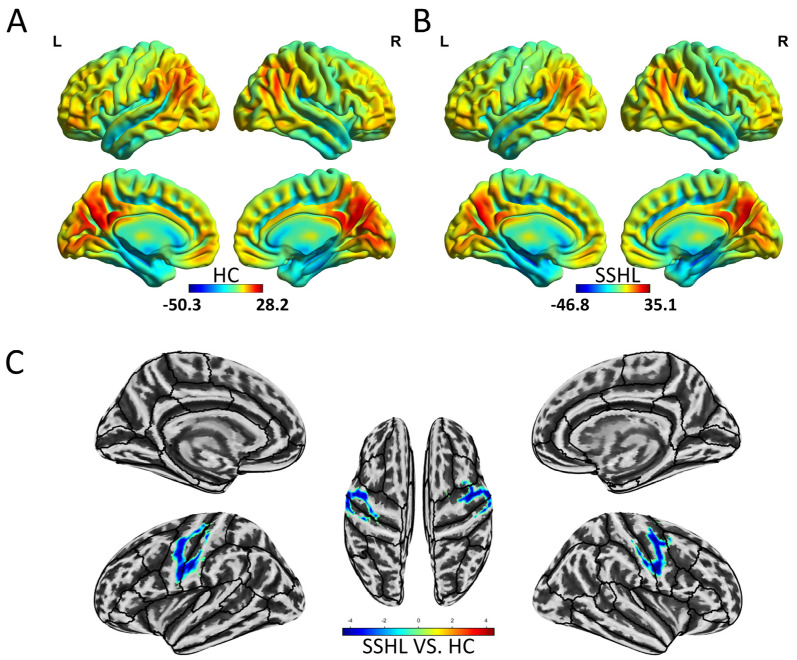
Altered static local brain connectivity in unilateral SSHL. (**A**) Within-group mean static ReHo maps in the HC. (**B**) Within-group mean static ReHo maps in the SSHL. (**C**) Group comparisons of static ReHo between the SSHL and HC groups (Gaussian random field correction; voxel-level *p* < 0.001, cluster-level *p* < 0.01). SSHL, sudden sensorineural hearing loss; HC, healthy controls; L, left; R, right.

Figure 2 illustrates the spatial distribution of the static ReHo in both the HC (Figure 2A) and SSHL (Figure 2B) groups. Compared to the HCs, SSHL patients exhibited significantly decreased static ReHo in the bilateral precentral gyrus, bilateral postcentral gyrus, and right middle frontal gyrus, along with increased static ReHo in the left posterior lobe of the cerebellum (GRF correction; voxel-level *p* < 0.001, cluster-level *p* < 0.01) (Figure 2C, Table 2).

### 3.2. Spatial Distribution and Group Differences of Dynamic ReHo

**Figure 3:**
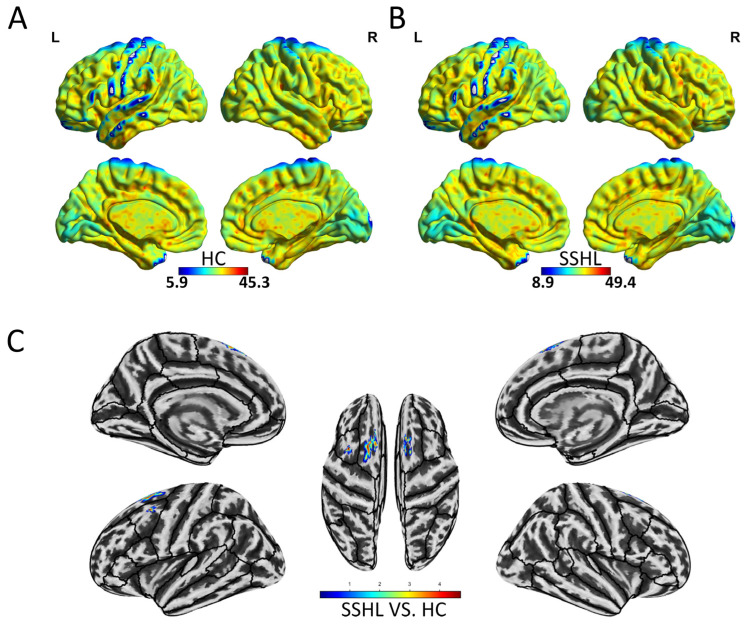
Altered dynamic local brain connectivity in unilateral SSHL. (**A**) Within-group mean dynamic ReHo maps in the HC. (**B**) Within-group mean dynamic ReHo maps in the SSHL. (**C**) Group comparisons of dynamic ReHo between the SSHL and HC groups (Gaussian random field correction; voxel-level *p* < 0.001, cluster-level *p* < 0.01). SSHL, sudden sensorineural hearing loss; HC, healthy controls; L, left; R, right.

Figure 3 depicts the spatial distribution of dynamic ReHo in both the HC (Figure 3A) and SSHL (Figure 3B) groups. Compared to the HCs, SSHL patients exhibited significantly increased dynamic ReHo in the bilateral middle frontal gyrus and bilateral superior frontal gyrus (GRF correction; voxel-level *p* < 0.001, cluster-level *p* < 0.01) (Figure 3C, Table 2).

### 3.3. Correlation Analysis

**Figure 4:**
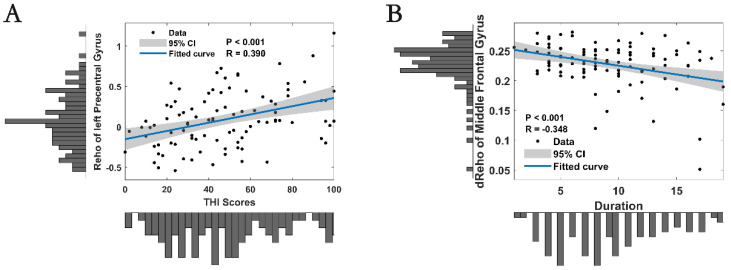
Clinical associations of altered static ReHo in SSHL patients. (**A**) Correlations between the THI scores and the static Reho of left precentral gyrus. (**B**) Correlations between the duration of SSHL and the dynamic Reho of middle frontal gyrus. SSHL, sudden sensorineural hearing loss; ReHo, regional homogeneity. Shaded areas represent 95% confidence intervals for the regression lines.

Correlation analyses revealed a positive correlation between static ReHo in the left precentral gyrus and THI scores (R = 0.39, *p* < 0.001, *n* = 102) (Figure 4A). Additionally, there was a negative correlation between dynamic ReHo in the middle frontal gyrus and the duration of hearing loss (R = −0.35, *p* < 0.001, *n* = 102) (Figure 4B).

### 3.4. Classification of SSHL Patients

**Figure 5:**
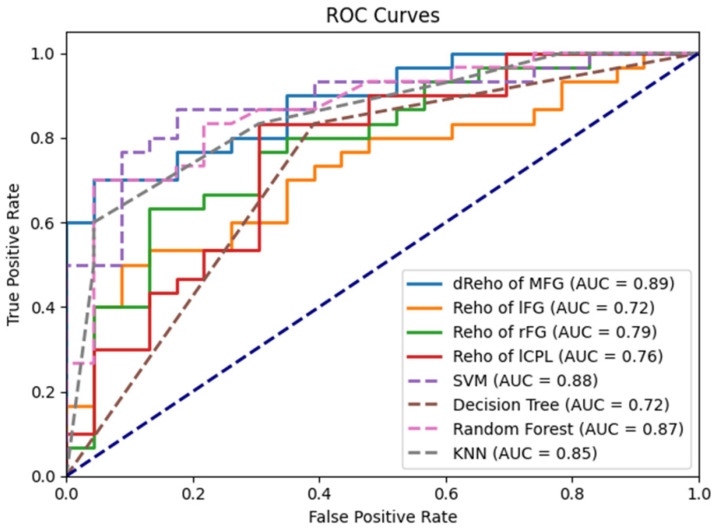
Receiver operating characteristic (ROC) curves for the classification of SSHL patients and healthy controls using dynamic ReHo features. Dashed lines represent the chance level (AUC = 0.5). ReHo, regional homogeneity; AUC, area under the curve.

The ROC analysis revealed a varying discriminatory power among individual neuroimaging features. The area under the ROC curve (AUC) values for each feature were as follows: dynamic ReHo of the middle frontal gyrus (dReHo of MFG) (AUC = 0.88), ReHo of the left frontal gyrus (lFG) (AUC = 0.72), ReHo of the right frontal gyrus (rFG) (AUC = 0.78), and ReHo of the left cerebellum posterior lobe (lCPL) (AUC = 0.76) (Figure 5). Logistic regression demonstrated robust performance across all features, with the dynamic ReHo of MFG showing the highest AUC.

Integrating all four features into predictive models using SVM, Decision Tree, Random Forest, KNN, and Logistic Regression resulted in varied performance outcomes. SVM achieved the highest AUC of 0.88, followed closely by Random Forest (AUC = 0.87). The Decision Tree and KNN models exhibited a moderate performance with AUCs of 0.72 and 0.85, respectively (Figure 5).

## 4. Discussion

In this study, we explored both static and dynamic ReHo in patients with unilateral SSHL and compared them to HCs. Our findings revealed unveiled substantial modifications in both static and dynamic ReHo in SSHL patients, indicating disrupted the local brain connectivity. Additionally, we found correlations between these ReHo changes and clinical characteristics, as well as the potential for ReHo metrics to serve as biomarkers for SSHL through ROC analysis.

Our analysis of static ReHo revealed significant decreases in the bilateral precentral gyrus, bilateral postcentral gyrus, and right middle frontal gyrus in SSHL patients compared to HCs. The precentral gyrus, which contains the primary motor cortex, plays a crucial role in voluntary motor control [14,33]. The observed decrease in ReHo in this area may reflect the disruption of motor functions related to auditory feedback mechanisms [34]. The postcentral gyrus, known for its involvement in somatosensory processing [35], may exhibit decreased ReHo due to the altered sensory integration resulting from auditory deficits [36,37]. The right middle frontal gyrus is associated with higher cognitive functions, including working memory and attention [38,39]. A decreased ReHo in this region could indicate cognitive impairment or compensation mechanisms due to the hearing loss [40,41]. These findings are consistent with previous studies that have identified changes in the motor and cognitive-related brain regions in SSHL patients [14,42,43].

Conversely, we observed an increase in static ReHo in the left posterior lobe of the cerebellum. The cerebellum is traditionally associated with motor control and coordination, but it also plays a role in cognitive functions and sensory processing [44,45]. The increase in ReHo in the cerebellum might reflect a compensatory mechanism aimed at maintaining motor and cognitive functions in the face of auditory impairment [35]. This may reflect the broader neural compensation of neural circuits in response to the loss of auditory input. Our findings align with the prior research suggesting cerebellar involvement in the adaptation to sensory deficits [15,46]. However, some studies have not reported such cerebellar changes, highlighting the need for further investigation into this brain region’s role in SSHL.

The static analysis of ReHo provides valuable insights into the baseline neural activity synchronization in brain regions, highlighting areas with a consistently altered connectivity in SSHL. However, it overlooks the dynamic fluctuations in brain activity that occur over time, which are crucial for understanding the adaptive responses and compensatory mechanisms in neurological disorders like SSHL [14,22,25]. By employing a dynamic ReHo analysis, our study aimed to capture these temporal variations, which are often obscured in static analyses. Dynamic ReHo metrics, such as variability and flexibility of regional synchronization patterns, offer a more nuanced view of how neural networks respond to auditory deficits in SSHL. This approach not only enhances our understanding of the temporal dynamics of brain function but also provides novel insights into the progressive changes and plasticity mechanisms that may underlie SSHL pathology [14,22].

The dynamic ReHo analysis showed significant increases in the bilateral middle frontal gyrus and bilateral superior frontal gyrus in SSHL patients. The increased dynamic ReHo in this region suggests a heightened temporal variability in cognitive processing, possibly as a response to the increased cognitive load associated with compensating for hearing loss [40]. The superior frontal gyrus is implicated in self-awareness and executive control [14,47]. The increased dynamic ReHo here may indicate an adaptive mechanism to maintain cognitive function and self-monitoring capabilities in the absence of normal auditory input [13,40]. These results extend previous static analyses by highlighting the importance of temporal dynamics in understanding brain function in SSHL [14,43].

While static and dynamic ReHo analyses both provide valuable insights into the brain connectivity alterations in SSHL, they capture different aspects of neural activity that may not always converge. Our study revealed notable discrepancies between the static and dynamic ReHo results, highlighting the importance of conducting dynamic ReHo analyses to complement the static findings [22]. The discrepancies between the static and dynamic ReHo results underscore the complementary nature of the dynamic ReHo analysis in understanding the full spectrum of brain connectivity alterations in SSHL [22,25]. The dynamic ReHo metrics capture transient changes and temporal dynamics that static measures may overlook, providing a more comprehensive view of how neural networks adapt and reorganize in response to sensory deprivation.

Correlation analyses further supported the clinical relevance of our ReHo findings. We identified a positive correlation between static ReHo in the left precentral gyrus and Tinnitus Handicap Inventory (THI) scores, indicating that a greater local connectivity in this region is associated with a higher severity. This suggests that increased motor cortex activity might be linked to the perception of tinnitus, possibly through maladaptive neuroplastic changes [12,48], potentially guiding non-invasive brain stimulation (e.g., rTMS) for modulation. This aligns with clinical trials demonstrating that rTMS reduces tinnitus severity [49,50,51]. Future studies could explore targeted rTMS protocols for SSHL patients with high precentral gyrus ReHo. Additionally, there was a negative correlation between dynamic ReHo in the middle frontal gyrus and the duration of hearing loss, suggesting that longer durations of SSHL are associated with reduced temporal variability in this region. This reduction in variability may reflect a decline in the brain’s ability to dynamically adapt to the chronic absence of auditory input. These correlation findings align with previous research showing links between altered brain activity and clinical symptoms in SSHL [14,43], but our study uniquely highlights the importance of dynamic brain changes.

The ROC analysis demonstrated that dynamic ReHo of the middle frontal gyrus (AUC = 0.88) had the highest discriminatory power among individual neuroimaging features, followed by ReHo of the right frontal gyrus (AUC = 0.78), the left cerebellum posterior lobe (AUC = 0.76), and the left frontal gyrus (AUC = 0.72). Clinically, this metric could facilitate the early identification of patients with altered neural plasticity, enabling timely interventions like cognitive–behavioral therapy or neuromodulation to enhance frontal lobe adaptability. While the current findings suggest a role for frontal lobe plasticity in SSHL, translating dynamic ReHo metrics into clinical practice requires validation through longitudinal trials of cognitive–neuromodulatory interventions. Integrating these features into predictive models using SVM, Decision Tree, Random Forest, KNN, and Logistic Regression showed that the SVM model achieved the highest AUC of 0.88, indicating its potential utility as a diagnostic tool for SSHL. The SVM classifier outperformed other models, likely due to its inherent ability to handle high-dimensional neuroimaging data through non-linear kernel functions, which effectively capture complex, non-linear relationships between dynamic ReHo features and SSHL status. Additionally, SVM’s regularization techniques mitigate overfitting by penalizing large model weights, ensuring robust generalization to unseen data. These properties make SVM particularly well-suited for decoding multi-voxel patterns in functional connectivity data, where subtle spatial–temporal features (e.g., frontal lobe dynamic ReHo variability) may distinguish SSHL patients from controls. Our findings corroborate earlier studies that have identified the potential of neuroimaging markers in diagnosing and understanding SSHL [13,14,15].

While this study highlights the utility of dynamic ReHo in capturing temporal brain dynamics, several limitations should be noted. First, the cross-sectional design does not allow for the determination of causal relationships between ReHo changes and SSHL. And our study focused on untreated SSHL patients in the acute phase, providing a snapshot of baseline neural dysregulation before the potential neuroplasticity induced by hearing restoration [52]. Longitudinal studies are needed to confirm these associations over time and to investigate how interventions like auditory training modulate the dynamic ReHo changes observed here. Second, although we included a relatively large sample size, the inherent heterogeneity of SSHL could still influence the results. Future studies should aim to replicate these findings in larger, more diverse cohorts. And we should acknowledge that the current sample size limits the power of laterality-based comparisons and advocate for larger studies to validate asymmetric neural responses. Third, static and dynamic ReHo metrics reflect distinct neural phenomena, and their divergent findings may complicate causal interpretation, as the static–dynamic decoupling could reflect independent pathological processes or measurement noise. Future studies should employ longitudinal designs to disentangle whether dynamic changes precede or follow static disruptions. Fourth, a notable limitation is the lack of a standardized assessment for subclinical emotional symptoms at the time of scanning. While participants were excluded if they had a history of psychiatric disorders, acute emotional responses to hearing loss could theoretically influence frontal lobe activity. Moreover, social isolation and reduced quality of life are well-documented consequences of SSHL [53,54], which can also independently affect frontal lobe activity and overall brain function. Future studies should incorporate standardized psychological assessments to dissociate the effects of SSHL from emotional comorbidities, particularly in chronic cohorts where emotional symptoms may be more prevalent. In addition, future research should consider measuring and controlling for social isolation and quality of life to provide a more comprehensive understanding of their impact on brain connectivity in SSHL. Finally, our study focused solely on ReHo metrics; incorporating other neuroimaging techniques such as functional connectivity and structural MRI could provide a more comprehensive understanding of brain alterations in SSHL.

In conclusion, our study demonstrates that SSHL is associated with significant alterations in both static and dynamic ReHo, with these changes correlating with clinical characteristics. The static ReHo analysis revealed significant disruptions in local synchronization within motor- and cognitive-related brain regions, indicative of baseline alterations in neural activity. Conversely, the dynamic ReHo analysis demonstrated increased temporal variability, particularly in frontal regions, suggesting adaptive responses to auditory deficits through neural plasticity. These findings highlight the complementary role of dynamic ReHo in capturing the nuanced changes in local connectivity that static measures may overlook. The potential of ReHo metrics as biomarkers for SSHL was supported by ROC analysis. Our study provides insights into the adaptive mechanisms underlying SSHL and underscores the potential for targeted therapeutic interventions aimed at enhancing local brain connectivity and contributes to advancing neuroimaging methods for medical research and holds promise for improving the assessment and treatment of SSHL patients.

## Figures and Tables

**Table 1:** Demographic and clinical variables.

Subjects	SSHL	HC	*p* Value
Number of subjects	102	73	N/A
Age (years)	38.89 ± 12.11	38.01 ± 16.52	0.685
Gender (male/female)	52/50	37/36	0.969
Education level (years)	12.67 ± 3.20	12.74 ± 2.93	0.878
Duration (days)	9.03 ± 4.27	N/A	N/A
Effected side (left/right)	56/46	N/A	N/A
PTA (dBHL)	73.66 ± 10.38	13.25 ± 5.08	<0.001
THI score	47.54 ± 26.37	N/A	N/A

Note. A Pearson chi-squared test was used for gender comparison. Independent-samples *t*-tests were used for age, education, and PTA comparisons. SSHL, sudden sensorineural hearing loss; HC, healthy control; PTA, pure tone average; THI, tinnitus handicap inventory. Duration: Time from symptom onset to MRI scanning.

**Table 2:** Comparison of static and dynamic ReHo between the SSHL and HC groups (voxel-level *p* < 0.001 and GRF corrected at cluster-level *p* < 0.01).

Brain Regions	Voxels	MNI Coordinates			*T* Values
		X	Y	Z	
Static ReHo (SSHL vs. HC)					
Left cerebellum posterior lobe	91	−15	−69	−30	4.1
Left precentral gyrus/Left postcentral gyrus	143	−57	−6	45	−4.6
Right precentral gyrus/Right postcentral gyrus/Right middle frontal gyrus	148	60	0	24	−4.5
Dynamic ReHo (SSHL vs. HC)					
Bilateral middle frontal gyrus, bilateral superior frontal gyrus	100	−27	9	66	5.6

MNI, Montreal Neurological Institute; SSHL, sudden sensorineural hearing loss; HC, healthy control; ReHo, regional homogeneity.

## Data Availability

Data is available on request due to ethical restrictions.
